# Surgeon anterior cruciate ligament reconstruction volume and rates of concomitant meniscus repair

**DOI:** 10.1186/s40634-023-00626-8

**Published:** 2023-06-08

**Authors:** Sahil Dadoo, Sean J. Meredith, Laura E. Keeling, Jonathan D. Hughes, Christopher Keenan, Mark Viecelli, James J. Irrgang, Bryson P. Lesniak, Volker Musahl

**Affiliations:** 1 Department of Orthopaedic Surgery, University of Pittsburgh Medical Center, Pittsburgh, PA USA; 2 Department of Orthopaedics, University of Maryland School of Medicine, Baltimore, MD USA; 3 University of Pittsburgh Medical Center, Pittsburgh, PA USA; 4 Department of Physical Therapy, University of Pittsburgh, Pittsburgh, PA USA

**Keywords:** ACL, Surgeon volume, Meniscus repair, Meniscectomy

## Abstract

**Purpose:**

The purpose of this study was to assess the effect of surgeon anterior cruciate ligament reconstruction (ACLR) volume on rates of ACLR with concomitant meniscus repair versus meniscectomy and subsequent meniscus surgeries.

**Methods:**

A retrospective review was conducted from a database of all ACLR performed between 2015 and 2020 at a large integrated health care system. Surgeon volume was categorized as < 35 ACLR per year (low-volume), and ≥ 35 ACLR per year (high-volume). Rates of concomitant meniscus repair and meniscectomy were compared between low-volume and high-volume surgeons. Subgroup analyses compared the rates of subsequent meniscus surgery and procedure time based on surgeon volume and meniscus procedure type.

**Results:**

A total of 3,911 patients undergoing ACLR were included. High-volume surgeons performed concomitant meniscus repair statistically significantly more often than low-volume surgeons (32.0% vs 10.7%, *p* < 0.001). Binary logistic regression indicated 4.15 times higher odds of meniscus repair among high-volume surgeons. Subsequent meniscus surgery occurred more commonly following ACLR with meniscus repair among low-volume surgeons (6.7% vs 3.4%, *p* = 0.047), but not high-volume surgeons (7.0% vs 4.3%, *p* = 0.079). Low-volume surgeons also had longer procedure times for concomitant meniscus repair (129.9 vs 118.3 min, *p* = 0.003) and meniscectomy (100.6 vs 95.9 min, *p* = 0.003).

**Conclusions:**

Data from this study shows that surgeons with lower volume of ACLR select meniscus resection statistically significantly more often than higher-volume surgeons. However, an abundance of literature is available to show that meniscus loss negatively affects the development of post-traumatic osteoarthritis in patients Therefore, as demonstrated in this study by high-volume surgeons, the meniscus should be repaired and protected whenever possible.

**Level of Evidence:**

III.

## Background

Studies have shown an association between increasing surgical volume and improved clinical outcomes. In the total hip arthroplasty literature, increasing surgeon volume is associated with greater implant survivorship, lower complication rates, shorter hospital length of stay, and lower costs [12, 19]. Total knee arthroplasty has similarly shown an association between surgeon volume and complications, revision rates, procedure times, transfusion rates, and patient-reported outcomes [15, 31]. The relationship between surgeon volume and outcomes is also reported in shoulder surgery, where a recent systematic review of 10 studies reported lower volume was associated with increased surgical complications, length of stay, surgical times, and costs [30].

Less is known about the relationship between surgeon anterior cruciate ligament (ACL) reconstruction (ACLR) volume and clinical outcomes. It has been reported that low surgeon ACLR volume is associated with greater operating room time, increased readmission rates, a greater risk of subsequent surgery, and greater costs of adverse events [9, 18]. Additionally, lower surgeon volume has been associated with increased allograft use during ACLR, compared to higher rates of patellar tendon autograft among high-volume surgeons [9]. Finally, high-volume surgeons have been shown to place more anatomic tunnels during ACLR, which may decrease the risk of ACL graft failure and revision ACLR [5, 8].

Concomitant meniscus injury is an important factor to consider in an ACL-deficient knee, as appropriate management of such injuries is crucial in restoring knee stability [25, 29]. Posterior horn medial meniscus tears have been associated with increased anterior tibial translation, while lateral meniscus posterior root tears have been associated with increased rotatory knee instability [1, 26]. Repair of meniscus tears has been shown to not only improve rotatory knee stability [6, 29], but also decrease the risk of post-traumatic osteoarthritis (OA), as meniscectomy has been associated with joint space narrowing and progression of articular cartilage damage after ACLR [10, 21]. As a result, meniscus repair has gained popularity as the primary treatment modality for traumatic meniscus tears in an effort to “save the meniscus” and preserve long-term knee stability and function [3, 24].

The purpose of this study was to assess the effect of surgeon ACLR volume on rates of concomitant meniscus repair versus meniscectomy, as well as subsequent meniscus surgeries. The hypothesis was that low-volume surgeons would perform concomitant meniscus repair less often than higher volume surgeons, and that low-volume surgeons would experience higher rates of subsequent meniscus surgery.

## Methods

This retrospective study was granted Institutional Review Board approval at the University of Pittsburgh (No: STUDY19030196). All patients that underwent primary ACLR at a large integrated health care system made up of both academic and community hospitals from January 2015 to December 2020 were identified from a registry database and analyzed for inclusion. Patients undergoing ACLR both with and without meniscus surgery were included. Exclusion criteria included patients < 14 years old, patients undergoing combined meniscus repair and meniscectomy, bilateral procedures at the time of primary ACLR, and multi-ligament reconstruction. The informatics system was queried for all consecutive ACLR cases performed over this time period based on CPT code, and concomitant meniscus surgeries were additionally recorded using CPT codes for meniscectomy and meniscus repair. Data was extracted from the system in January 2023, allowing for minimum 2-year follow-up to assess for subsequent meniscus surgeries.

Data regarding patient age, sex, and body mass index (BMI), as well as performing surgeon, were recorded. Low surgeon volume was defined as performing fewer than 35 ACLR per year, and high surgeon volume defined as performing 35 or more ACLR per year, based on previous literature demonstrating an increased risk for subsequent knee surgery among surgeons performing < 35 ACLR per year [8, 23]. Surgeon yearly ACLR volume was averaged over all active years of practice within the time period of the study to classify each surgeon.

The primary outcome was the rate of concomitant meniscus repair versus meniscectomy performed at the time of primary ACLR, which was compared between low- and high-volume surgeon groups. All patients undergoing meniscus surgery were further evaluated for rates of subsequent meniscus surgeries and primary ACLR procedure time based on surgeon volume and meniscus procedure type (meniscectomy versus meniscus repair).

Statistical analysis was performed using chi-square tests to compare categorical variables, and independent t-tests or Mann–Whitney U tests for parametric and non-parametric continuous variables, respectively, between low-volume and high-volume surgeon groups. Binary logistic regression was performed to calculate odds ratios (OR) and 95% confidence intervals (95% CI) for significant findings. Statistical significance was set at *p* < 0.05.

## Results

**Fig. 1:**
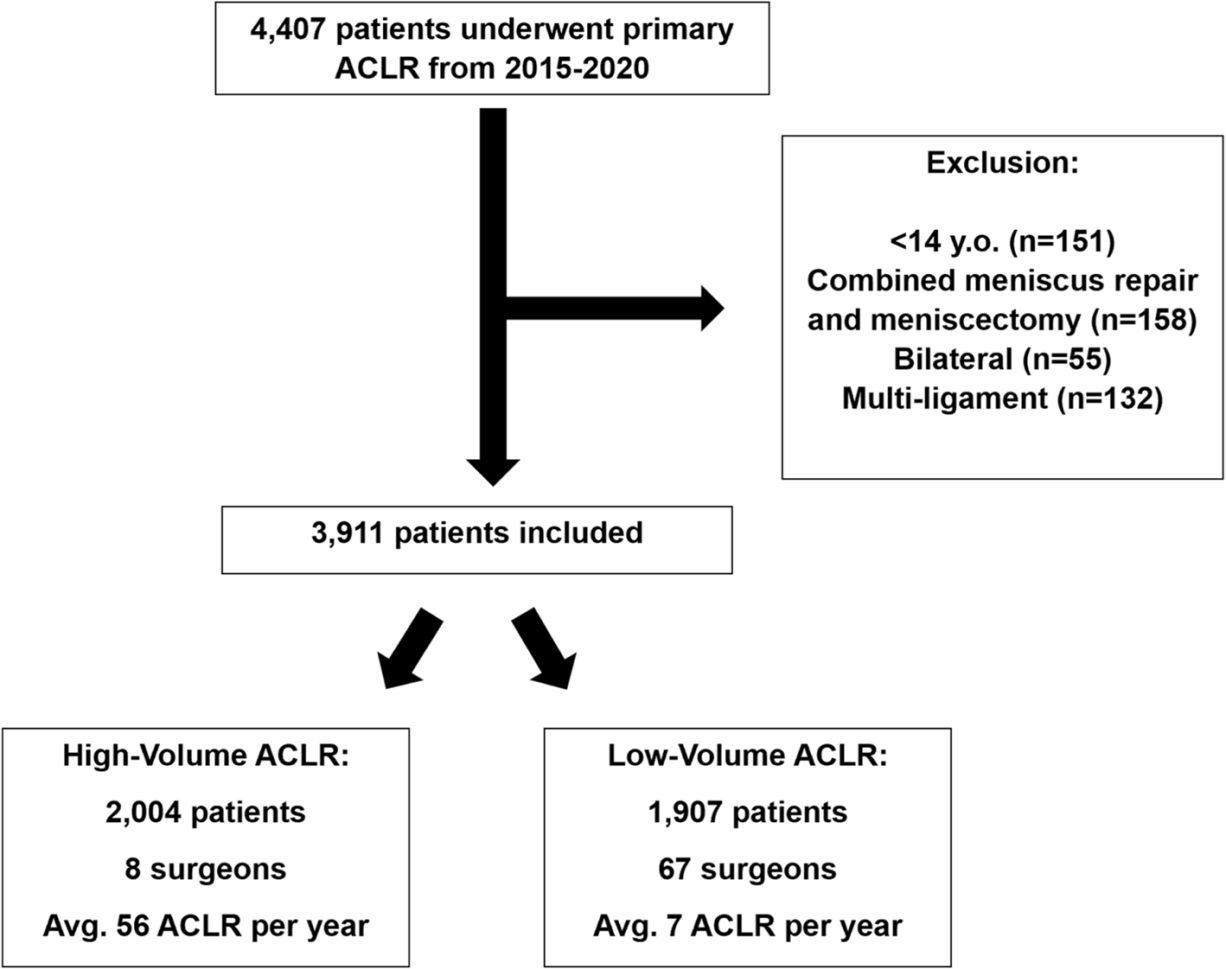
Flowchart of Inclusion and Exclusion Criteria. ACLR = anterior cruciate ligament reconstruction

Between 2015 and 2020, a total of 4,407 patients underwent primary ACLR. Following application of exclusion criteria, 3,911 patients were included, of which 2,004 patients were operated on by 8 high-volume surgeons and 1,907 patients were operated on by 67 low-volume surgeons (Fig. 1). High-volume surgeons performed an average of 56 ACLR per year, whereas low-volume surgeons performed an average of 7 ACLR per year.

The mean age of patients undergoing ACLR was 26.7 years (SD: 11.6 years; range: 14 to 64 years), and 44.8% of patients were female. The mean BMI of the cohort was 27.5 kg/m^2^ (SD: 5.9 kg/m^2^). The mean age of patients in the low-volume surgeon group was significantly higher than the high-volume surgeon group (28.8 vs 24.8 years; *p* < 0.001). Additionally, patients in the low-volume cohort had a higher BMI than the high-volume cohort (28.4 vs 26.6 kg/m^2^; *p* < 0.001) (Table 1). No differences in rates of subsequent meniscus surgeries were identified between surgeon volume groups for the total cohort (Table 1).

**Table 1:** Patient demographics and rates of subsequent meniscus surgeries based on surgeon volume

	**Total cohort (*N* = 3911)**	High-volume surgeon group* (*N* = 2004)	Low-volume surgeon group* (*N* = 1907)	*p*-value*
Age (years)	26.7 +/- 11.6	24.8 +/- 10.6	28.8 +/- 12.2	**< *0.001***
Sex				0.509
Female, N (%)	1755 (44.8)	889 (44.4)	860 (45.1)	
Male, N (%)	2156 (55.1)	1115 (55.6)	1041 (54.6)	
BMI (kg/m^2^)	27.5 +/- 5.9	26.6 +/- 5.4	28.4 +/- 6.2	**< *0.001***
Subsequent meniscus surgeries, N (%)	149 (3.8)	86 (4.3)	63 (3.3)	0.107

Reported as means +/- standard deviation or N with percentages

*BMI* Body mass index

^*^Chi-square and Mann–Whitney U test performed. *p*-values assessing high volume versus low volume groups. Significance set as *p* < 0.05 (bold)

Among the 3,911 patients included in the study, ACLR with concomitant meniscus repair was performed in 835 cases (21.4%), and ACLR with concomitant meniscectomy was performed in 899 cases (23.0%). Isolated ACLR without meniscus surgery was performed in 2,177 cases (55.7%), and was more prevalent among low-volume surgeons compared to high-volume surgeons (62.7% vs 49.5%; *p* < 0.001) (Table 2). High-volume surgeons performed concomitant meniscus repair statistically significantly more often (32.0% vs 10.2%; *p* < 0.001), and concomitant meniscectomy less often (18.6% vs 27.6%; *p* < 0.001), than low-volume surgeons (Table 2). Binary logistic regression indicated that high-volume surgeons had 4.15 times higher odds of performing meniscus repair versus meniscectomy when compared to low-volume surgeons (OR: 4.15; 95% CI: 3.48–4.95; *p* < 0.001) (Table 3).

**Table 2:** Comparison of concomitant meniscus surgery during primary ACLR based on surgeon volume

	**Total cohort (*N* = 3911)**	High-Volume Surgeons* (*N* = 2004)	Low-Volume Surgeons* (*N* = 1907)	*P* *
ACL without meniscus surgery, N (%)	2177 (55.7)	991 (49.5)	1186 (62.2)	**< *0.001***
ACL with meniscus repair, N (%)	835 (21.4)	641 (32.0)	194 (10.2)	**< *0.001***
ACL with meniscectomy, N (%)	899 (23.0)	372 (18.6)	527 (27.6)	**< *0.001***

Reported as N with percentages

*ACL* Anterior cruciate ligament, *ACLR* Anterior cruciate ligament reconstruction

^*^Chi-square test performed. *p*-values assessing high-volume versus low-volume groups. Significance set as *p* < 0.05 (bold)

**Table 3:** Binary logistic regression for meniscus repair versus meniscectomy

	**Meniscus Repair versus Meniscectomy**^**a**^		
Variable	**B (SE)**^**b**^	**Odds Ratio (95% CI)**^**c**^	***p*-value**
Constant	-2.18 (0.08)	0.11	**< *0.001***
High-Volume Surgeon (baseline: low-volume)	1.42 (0.09)	4.15 (3.48–4.95)	**< *0.001***

Binary logistic regression demonstrating influence of high-volume surgeon on rate of meniscus repair versus meniscectomy when compared to low-volume surgeon

^a^ Nagelkerke R^2^ = 0.111

^b^ B = regression coefficient, SE = standard error

^c^ 95% CI = 95% confidence interval

In the total study population, subsequent meniscus surgery occurred more commonly following ACLR with meniscus repair versus meniscectomy (6.9% vs 3.8%; *p* = 0.003). However, when stratified based on surgeon volume, the increased rate of subsequent meniscus surgery following meniscus repair versus meniscectomy was apparent among low-volume surgeons (6.7% vs 3.4%, *p* = 0.047), but not high-volume surgeons (7.0% vs 4.3%, *p* = 0.079) (Table 4). Additionally, increased procedure times were found in the low-volume surgeon group compared to the high-volume group across all procedure types (106.5 vs 103.2 min; *p* = 0.002), and when stratified by ACLR with concomitant meniscus repair (129.9 vs 118.8 min; *p* = 0.003) and meniscectomy (100.6 vs 95.9 min; *p* = 0.003) (Table 5).

**Table 4:** Subsequent meniscus surgery based on surgeon volume and meniscus procedure type

	**Total Cohort (*N* = 3911)**	ACL with meniscus repair* (***N*** **= 835)**	ACL with meniscectomy* (*N* = 899)	*p*-value*
Subsequent Meniscus, N (%)	149 (3.8)	58 (6.9)	34 (3.8)	***0.003***
**High-Volume Surgeons**	**Total Cohort (*N* = 2004)**	ACL with meniscus repair* (***N*** **= 641)**	ACL with meniscectomy* (*N* = 372)	*p*-value*
Subsequent Meniscus, N (%)	86 (4.3)	45 (7.0)	16 (4.3)	0.079
**Low-Volume Surgeons**	**Total Cohort (*N* = 1907)**	ACL with meniscus repair* (***N*** **= 194)**	ACL with meniscectomy* (*N* = 527)	*p*-value*
Subsequent Surgery, N (%)	63 (3.3)	13 (6.7)	18 (3.4)	***0.047***

Reported as N with percentages

*ACL* Anterior cruciate ligament

^*^Chi-square test performed. p-values assessing meniscus repair versus meniscectomy groups. Significance set as *p* < 0.05 (bold)

**Table 5:** Procedure time based on surgeon volume and meniscus procedure type

**All Procedures**	**Total Cohort (*N*** **= 3911)**	**High-Volume Surgeons (*N*** **= 2004)**	**Low-Volume Surgeons (*N*** **= 1907)**	*p*-value*
Procedure Time (min), mean (SD)	104.9 (39.7)	103.2 (39.9)	106.5 (39.4)	***0.002***
**Meniscus Repair**	**Total Cohort (*N* = 835)**	**High-Volume Surgeons (*N* = 641)**	**Low-Volume Surgeons (*N* = 194)**	*p*-value*
Procedure Time (min), mean (SD)	121.0 (36.2)	118.3 (33.1)	129.9 (43.7)	***0.003***
**Meniscectomy**	**Total Cohort (*N* = 899)**	**High-Volume Surgeons (*N* = 372)**	**Low-Volume Surgeons (*N* = 527)**	*p*-value*
Procedure Time (min), mean (SD)	98.7 (33.8)	95.9 (34.8)	100.6 (33.0)	***0.003***

Reported as means +/- standard deviation

^*^Chi-square test performed. *p*-values assessing meniscus repair versus meniscectomy groups. Significance set as *p* < 0.05 (bold)

## Discussion

The most important finding of this study was that low-volume surgeons performed ACLR with concomitant meniscus repair at a significantly lower rate than high-volume surgeons. Additionally, concomitant meniscus repair resulted in an increased rate of subsequent meniscus surgery among low-volume surgeons, but not high-volume surgeons. This is one of few studies to assess the impact of surgeon ACLR volume on rates of meniscus repair versus meniscectomy, and uniquely analyzes a large, heterogeneous cohort from both academic and community physicians.

The primary finding of this study was consistent with two previous studies on ACLR surgeon volume [16, 32]. A cross-sectional study of a community-based ACLR registry reported surgeons performing 52 or more ACLR per year were 1.68 times more likely to perform a concomitant meniscus repair than surgeons performing between 6 and 52 ACLR per year, and 2.56 times more likely than surgeons performing fewer than 6 ACLR per year [32]. Another study of a statewide surgical database reported that surgeons performing more than 25 ACLR per year were 1.196 times more likely to perform a concomitant meniscus repair than lower volume surgeons [16]. In contrast, an analysis of the American Board of Orthopaedic Surgeons database from 2003 to 2007 did not find a difference in rates of concomitant meniscus repair between those performing fewer than 5 ACLR per year and more than 26 ACLR per year [20]. Instead, sports medicine fellowship training was associated with performing concomitant meniscus repair as compared to other fellowship training or no fellowship training. Given the conflicting data in the literature, the present study adds a very large sample size that supports prior literature demonstrating an association between higher surgeon ACLR volume and concomitant meniscus repair. Specifically, our study identifies high surgeon ACLR volume as a significant predictor of performing meniscus repair versus meniscectomy, with 4.15 times increased odds when compared to low surgeon ACLR volume. This is a clinically significant finding when considering recent trends that favor meniscus repair over meniscectomy in an effort to preserve the long-term health of the knee joint [2, 14, 28].

In our study, an increased rate of subsequent meniscus surgery was additionally found following meniscus repair versus meniscectomy by low-volume surgeons, but not high-volume surgeons. Previous literature has shown meniscus repair results in higher rates of subsequent meniscus surgery when compared to meniscectomy [11], with a recent systematic review indicating a 19% failure rate up to 6 years following meniscus repair [22]. However, the importance of meniscal repair cannot be understated [3] due to the unfavorable long-term outcomes following meniscectomy versus meniscus repair, such as increased progression of OA (60% versus 20%) and lower return to pre-injury activity level (50% versus 96.2%) at mean 8.8 years follow-up [27]. In addition, more recent literature has demonstrated lower rates of subsequent meniscectomy following meniscus repair, likely due to advances in surgical techniques and devices [17]. Further, in the setting of ACLR, meniscus repair has the potential to limit post-traumatic OA given that meniscectomy with concomitant ACLR has been associated with joint space narrowing and articular cartilage damage [10, 21]. While concomitant ACLR has been shown to be protective with regard to subsequent meniscus surgery following meniscus repair [13], patients treated by high-volume surgeons are at further decreased risk of subsequent knee surgery compared to patients treated by low-volume surgeons [17]. Our findings mirror prior literature demonstrating a higher rate of subsequent meniscus surgery following meniscus repair by a low-volume ACLR surgeon [16, 17], whereas no such finding was apparent amongst high-volume ACLR surgeons in our study.

Finally, an increased procedure time was observed among low-volume surgeons compared to high-volume surgeons in the setting of meniscus repair and meniscectomy. Previous literature has demonstrated that lower volume surgeons have increased primary ACLR procedure times compared to high-volume surgeons, especially in the tendon harvesting phase [7]. It is therefore possible that the increased procedure times among low-volume surgeons in our study were a result of the ACLR procedure as opposed to the meniscus procedure performed. Despite this, there are important adverse effects of prolonged procedure times in the setting of ACLR, and surgeon volume may play an important role based on previous literature [4, 9]. A recent large registry study of 12,077 ACLR found increased procedure time to be independently associated with increased rates of overnight hospital stay, hospital readmission, return to the operating room, and 30-day complication rates [4]. Further, a review of multiple statewide databases showed surgeons performing fewer than 6 ACLR per year had greater operating room times and higher rates of non-routine discharges resulting in increased resource utilization [9]. While the effect of surgeon volume on meniscus procedure times has not been identified in the literature, the increased procedure times observed among low-volume surgeons in our study are an important consideration for patients undergoing ACLR with concomitant meniscus tears.

There are several limitations to the present study. First, as a large registry study, specific data regarding the type, location, and size of concomitant meniscus tears remains unknown, which may impact the decision to perform meniscus repair versus meniscectomy. However, the large sample size of our study increases the likelihood of evenly distributed meniscus tear patterns. Second, there were significant age and BMI differences between the surgeon volume groups, which may have impacted the decision to perform meniscus repair versus meniscectomy, and influenced rates of subsequent meniscus surgery. However, while statistically significant, the clinical difference in these variables was small, and the potential impact of such differences is likely minimal when considering the significant difference in the rates of meniscus repair and meniscectomy between surgeon volume groups. Finally, as a retrospective registry review of a single large healthcare system, patients who sought care outside of our healthcare system were lost to follow-up, which has the potential to confound our results, specifically regarding rates of return meniscus procedures. However, the large sample size of our study again favors the likelihood that loss of follow-up was evenly distributed between surgeon volume groups, and the increased rate of subsequent meniscus surgery following meniscus repair by low-volume surgeons remains an important finding of the study.

The findings of this study suggest that high-volume surgeons perform ACLR with concomitant meniscus repair significantly more often than low-volume surgeons. Our results complement previous literature and provide increased evidence on the relationship between surgeon volume and rates of meniscus repair versus meniscectomy. This data may be utilized by surgeons to highlight differences in care based on surgeon characteristics, in an effort to discover avenues for change and optimize patient outcomes following ACLR with concomitant meniscus pathology.

## Conclusions

Low-volume surgeons were observed to perform ACLR with concomitant meniscus repair statistically significantly less often than high-volume surgeons. Further, low-volume surgeons demonstrated a higher rate of subsequent meniscus surgery following meniscus repair versus meniscectomy, whereas no significant difference was found among high-volume surgeons. An abundance of literature is available to show that meniscus loss negatively affects the development of post-traumatic osteoarthritis in patients, and therefore, as demonstrated in this study by high-volume surgeons, the meniscus should be repaired and protected whenever possible.

## Data Availability

No publicly available data was used for this study. Data sharing is not applicable to this article.
